# Patterns of care for prostate cancer radiotherapy—results from a survey among German-speaking radiation oncologists

**DOI:** 10.1007/s00066-020-01738-1

**Published:** 2021-01-27

**Authors:** Marco M. E. Vogel, Sabrina Dewes, Eva K. Sage, Michal Devecka, Jürgen E. Gschwend, Kilian Schiller, Stephanie E. Combs

**Affiliations:** 1 Department of Radiation Oncology, Klinikum rechts der Isar, Technical University of Munich (TUM), Ismaninger Straße 22, 81675 Munich, Germany; 2 Institute for Radiation Medicine (IRM), Department of Radiation Sciences (DRS), Helmholtz Zentrum München, Neuherberg, Germany; 3 Department of Urology, Klinikum rechts der Isar, Technical University of Munich (TUM), Munich, Germany; 4 Partner Site Munich, Deutsches Konsortium für Translationale Krebsforschung (DKTK), Munich, Germany

**Keywords:** Prostate carcinoma, Treatment pathways, Questionnaire, German-speaking countries, Radiation therapy

## Abstract

**Background:**

Emerging moderately hypofractionated and ultra-hypofractionated schemes for radiotherapy (RT) of prostate cancer (PC) have resulted in various treatment options. The aim of this survey was to evaluate recent patterns of care of German-speaking radiation oncologists for RT of PC.

**Methods:**

We developed an online survey which we distributed via e‑mail to all registered members of the German Society of Radiation Oncology (DEGRO). The survey was completed by 109 participants between March 3 and April 3, 2020. For evaluation of radiation dose, we used the equivalent dose at fractionation of 2 Gy with α/β = 1.5 Gy, equivalent dose (EQD2 [1.5 Gy]).

**Results:**

Median EQD2(1.5 Gy) for definitive RT of the prostate is 77.60 Gy (range: 64.49–84.00) with median single doses (SD) of 2.00 Gy (range: 1.80–3.00), while for postoperative RT of the prostate bed, median EQD2(1.5 Gy) is 66.00 Gy (range: 60.00–74.00) with median SD of 2.00 Gy (range: 1.80–2.00). For definitive RT, the pelvic lymph nodes (LNs) are treated in case of suspect findings in imaging (82.6%) and/or according to risk formulas/tables (78.0%). In the postoperative setting, 78.9% use imaging and 78.0% use the postoperative tumor stage for LN irradiation. In the definitive and postoperative situation, LNs are irradiated with a median EQD2(1.5 Gy) of 47.52 Gy with a range of 42.43–66.00 and 41.76–62.79, respectively.

**Conclusion:**

German-speaking radiation oncologists’ patterns of care for patients with PC are mainly in line with the published data and treatment recommendation guidelines. However, dose prescription is highly heterogenous for RT of the prostate/prostate bed, while the dose to the pelvic LNs is mainly consistent.

**Supplementary Information:**

The online version of this article (10.1007/s00066-020-01738-1) contains supplementary material, which is available to authorized users.

## Introduction

Radiotherapy (RT) for prostate cancer (PC) has changed over the past years. Whereas in the past conventionally fractionated RT was the standard of care, today, moderately hypofractionated schemes using data from the CHiPP trial, HYPRO trial, PROFIT trial, and Lee et al. are emerging [1–4]. Ultra-hypofractionated RT for PC is still under evaluation, since the results of the recently published PACE‑B trial are promising [5].

Further, the question of whether or not to treat pelvic lymph nodes (LNs) is still important. For definitive RT, studies have shown no benefit for RT of the pelvic LNs in cN0 situations [6–9]. However, if there is suspicion of LN involvement, treatment is usually administered.

For adjuvant RT, Abdollah et al. showed an advantage for certain groups [10], while the recently published SPPORT trial (NRG Oncology/RTOG 0534) showed a benefit for additional LN irradiation in the salvage situation [11]. Overall, LN irradiation in definitive and postoperative settings is under discussion and is handled inconsistently.

Overall, in clinical routine, treatment reality can differ according to the available data and guidelines. Therefore, this survey aims at evaluating the recent patterns of care for definitive and postoperative RT of PC of German-speaking radiation oncologists, to determine if they are in line with the published data and treatment guidelines and to learn whether emerging fractionations have already arrived in daily practice.

## Materials and methods

Experienced radiation oncologists developed a questionnaire with 35 items on RT planning with/without PSMA-PET imaging for treatment of PC. Questions were created as single-choice questions, multiple-choice questions, or free-response questions. A team of radiation oncologists and specialists in nuclear medicine reviewed the survey and applied minor changes to enhance usability and readability. For distribution of the questionnaire, we used the online platform survio.com. We sent a hyperlink via e‑mail to all registered members of the German Society of Radiation Oncology (DEGRO). Participation was voluntary as well as anonymous and available for completion between March 3 and April 3, 2020. The first part of the survey is analyzed in the present manuscript focusing on daily practice patterns with special focus on dose prescriptions, as well as the integration of novel fractionation regimes into daily practice. All aspects focusing on the use of molecular imaging in PC will be analyzed separately and are not part of the present manuscript.

When participants answered questions concerning total doses and single doses (SD) with ranges, we chose the lower end for analysis. We excluded dose values if total dose and SD did not match. For evaluation of the doses, we used the equivalent dose at fractionation of 2 Gy (EQD2), calculated using the linear quadratic model with α/β prostate = 1.5 Gy, i.e., EQD2(1.5 Gy). Conventionally fractioned RT was defined as a SD of 1.8–2.0 Gy/fraction, while moderate hypofractionation was defined as a SD of >2.0–4.00 Gy/fraction. We calculated dependencies for nominal variables with a chi-square test. All statistical analysis was performed using SPSS version 25 (IBM, Armonk, NY, USA).

## Results

A total of 109 participants completed the survey. The characteristics of participants are shown in Table 1.

**Table 1:** Characteristics of participants (*n* = 109)

	*n* (%)
*Participants’ institution*	
University hospital	29 (26.6)
Non-university hospital	26 (23.9)
Ambulatory health care center	37 (33.9)
Medical practice	17 (15.6)
*Participants’ position*	
Resident	10 (9.2)
Fellow/specialist	45 (41.3)
Leading medical personnel	54 (49.5)
*Available RT techniques*	
3D-CRT	100 (91.7)
IMRT/VMAT	108 (99.1)
Helical IMRT	26 (23.9)
IGRT	97 (89.0)
Stereotactic RT	80 (73.4)
Proton/heavy ion RT	2 (1.8)
Brachytherapy	64 (58.7)

*3D-CRT* three-dimensional conventional radiotherapy, *IMRT* intensity-modulated radiotherapy, *VMAT* volumetric arc therapy, *IGRT* image-guided radiotherapy, *RT* radiotherapy

### Definitive radiotherapy

Most radiation oncologists use in-house standard operating procedures (SOP) for target delineation (72.5%, 79/109), followed by EORTC (67.0%, 73/109) guidelines [12], and ESTRO ACROP (48.6%, 53/109) guidelines [13] (individual delineation: 8.3%, 9/109; RTOG protocol [14]: 1.8%, 2/109; other 0.9%, 1/109).

**Fig. 1:**
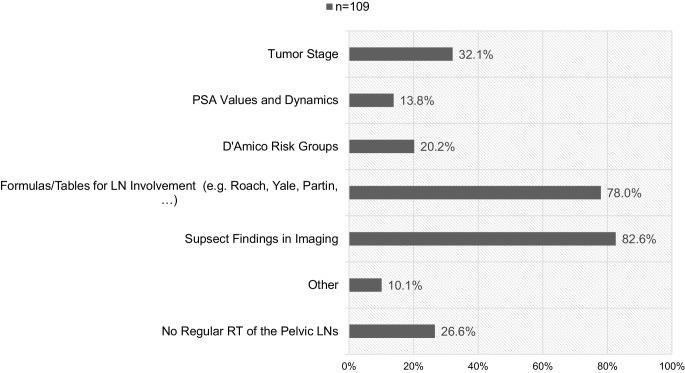
Factors and reasons for additional radiotherapy of the pelvic lymph nodes (*LNs*) in cases of definitive radiotherapy (*RT*; *n* = 109, multiple choices possible). *PSA *prostate-specific antigen

Of the participants, 65.1% (71/107) use magnetic resonance imaging (MRI) as a standard to plan definitive RT in patients with PC. Ambulatory institutions (medical practices and ambulatory health care centers) use MRI in 53.7% (29/54) and non-ambulatory institutions (university and non-university hospitals) in 76.4% (42/55). The chi-square test showed that ambulatory institutions use MRI significantly less than non-ambulatory institutions (*p* = 0.01). Fig. 1 shows factors and reasons for additional RT to the pelvic LNs. Median total dose of the prostate is 76.00 Gy (range: 60.00–84.00 Gy) with median SDs of 2.00 Gy (range: 1.80–3.00 Gy), which translates to an EQD2(1.5 Gy) of 77.60 Gy (range: 64.49–84.00 Gy). Median total dose to the pelvic LNs is 50.40 Gy (range: 44.00–66.30 Gy) in median SDs of 1.80 Gy (range: 1.60–2.00 Gy), which translates into an EQD2(1.5 Gy) of 47.52 Gy (range: 42.43–66.00 Gy). Table 2 shows the doses radiation oncologists will prescribe for definitive RT of the prostate and pelvic LNs.

**Table 2:** Median doses for definitive RT and postoperative RT as well as pelvic lymph nodes

	Median total dose in EQD2(1.5 Gy) [Gy]	Median single dose [Gy]	*n* = 109
*Definitive RT of prostate*	77.76 (range: 64.49–84.00)	2.00 (range: 1.80–3.00)	Conventional FX: 79 (72.5%) Moderate HFX: 20 (18.3%) Missing: 9 (8.3%) Brachytherapy: 1 (0.9%) with 145 Gy
*Definitive RT of pelvic LNs*	47.52 (range: 42.43–66.00)	1.80 (range 1.60–2.00)	Conventional FX: 107 (98.2%) Moderate HFX: 0 (0%) Missing: 2 (1.8%)
*Postoperative RT of prostate bed*	66.00 (range: 60.00–74.00)	2.00 (range: 1.80–2.00)	Conventional FX: 106 (97.2%) Moderate HFX: 0 (0%) Missing: 3 (2.8%)
*Postoperative RT of pelvic LNs*	47.52 (range: 41.76–62.79)	1.80 (range: 1.40–2.00)	Conventional FX: 107 (98.2%) Moderate HFX: 0 (0%) Missing: 2 (1.8%)

*RT* = radiotherapy, *LN* = lymph nodes, (*H)FX* = (hypo)fractionation, *EQD2(1.5* *Gy)* equivalent dose at fractionation of 2 Gy with α/β = 1.5 Gy

According to National Comprehensive Cancer Network (NCCN) risk groups, most participants recommend ADT in addition to definitive RT for unfavorable intermediate risk (74.3%, 81/109) or higher. 0.9% (1/109) and 9.2% (10/109) recommend ADT for patients with low risk or higher and favorable intermediate risk or higher, respectively. The participants will exclusively prescribe ADT for high-risk PC or higher and very high risk in 14.7% (16/109) and 0.9% (1/109) of the cases.

### Postoperative radiotherapy

**Fig. 2:**
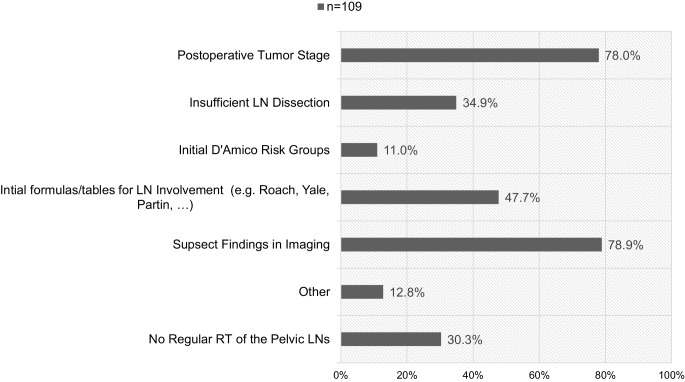
Factors and reasons for additional radiotherapy (*RT*) of the pelvic lymph nodes (*LNs*) in cases of postoperative radiotherapy (*n* = 109, multiple choices possible)

While 71.6% (78/109) of participants use in-house SOPs for target delineation of the postoperative prostate bed (PB), 67.0% (73/109) and 55.0% (60/109) use RTOG [15] and EORTC guidelines [16], respectively. 6.4% (7/109) of the participants do not use any guidelines for delineation. 3.7% (4/109) use the PMH guideline [17] and 1.8% (2/109) use the FROGG-RANZCR [18] guideline. Median total dose of the PB is 66.60 Gy (range: 60.00–74.00 Gy) with median SDs of 2.00 Gy (range: 1.80–2.00 Gy), which translates to an EQD2(1.5 Gy) of 66.00 Gy (range: 60.00–74.00 Gy). Median total dose of the pelvic LNs is 50.40 Gy (range: 44.00–66.60 Gy) in median SDs of 1.80 Gy (range: 1.40–2.00 Gy), which translates into an EQD2(1.5 Gy) of 47.52 Gy (range: 41.76–62.79 Gy). Table 2 shows the stated doses for postoperative RT of the PB and pelvic LNs and Fig. 2 shows factors and reasons for additional RT to the pelvic LNs.

For patients who receive salvage RT, 74.3% (81/109) of the participants will recommend ADT if risk factors are present. 18.3% (20/109) will never recommend additional ADT, while 7.3% (8/109) recommend ADT for all patients receiving salvage RT. The participants will recommend additional ADT for a median time of 12 months (range: 2–36 months) and a mean time of 15.3 months (standard deviation: 8.9 months). Table 3 shows the duration of ADT in groups.

**Table 3:** Additional ADT to salvage RT (*n* = 89)

	*n* (%)
*Additional ADT to salvage RT*	
≤6 months	31 (34.8)
7–12 months	15 (16.9)
13–24 months	40 (44.9)
>24 months	3 (3.4)

*ADT* androgen deprivation therapy, *RT* radiotherapy

Of all participants, 69.7% (76/109) will perform in-field re-RT in case of previous postoperative RT and 65.1% (71/109) in case of previous definitive RT. 27.5% will not perform re-RT.

## Discussion

We conducted a multi-center survey among German-speaking radiation oncologists concerning patterns of care for definitive and postoperative RT (adjuvant or salvage RT) for PC.

For definitive RT, most radiation oncologists use in-house SOPs, followed by the EORTC [12] and the ESTRO ACROP [13] guidelines. The RTOG [14] protocols are consulted by two participants. The main difference between those guidelines is the extent of inclusion of the seminal vesicles (SV) into the clinical target volume (CTV). The EORTC guideline recommends inclusion of the proximal SV of 1 cm for intermediate and of 2 cm for high-risk PC [12]. The RTOG-0815 protocol only recommends the inclusion of 1 cm of SV [14]. Qi et al. compared the EORTC guideline, the RTOG-0815 protocol, and actual anatomy and proposed 1.4 cm of SV for intermediate-risk PC and 2.2 cm of SV for high-risk PC, as they found the inclusion of 1 to 2 cm inadequate [12]. This was implemented in the recent ESTRO ACROP guidelines [13].

Two thirds of the radiation oncologists use MRI for treatment planning of definitive RT, with a significant higher proportion among non-ambulatory institutions. The delineation of the prostate is typically based on CT. However, MRI is usually superior in imaging of the soft tissue aspects. Steenbakkers et al. even showed that the dose to the rectal wall and penis bulb is significantly reduced by MRI-based RT planning [19]. However, radiation oncologists working in ambulatory institutions seem to use MRI less than their non-ambulatory colleagues. This might be owed to the fact that some ambulatory facilities do not have access to MRI.

The median EQD2(1.5 Gy) for definitive RT of the prostate is 77.76 Gy (range 69.29–84.00 Gy). This is in line with the recommendation of 74 to 80 Gy of the European [20] and German [21] guidelines. Goldner et al. previously presented data of Austrian radiation oncologists from 2007 and showed that the dose applied for definitive RT ranged from 70 to 78 Gy [22]. Our survey showed a highly heterogenous dose prescription. Only 18.3% of the participants use hypofractionated schemes, although recent trials [1–4] showed that moderate hypofractionation is feasible. Some authors even opt for a risk-adapted moderate hypofractionation [23]. The majority of participants (72.5%) still use conventional fractionation. This might be owed to the fact that the data on toxicity is heterogenous among the four trials, with an overall higher acute toxicity for moderately hypofractionated RT [1–4]. The lower use might also be explained by the German guideline [21] being cautious to recommend moderately hypofractionated RT, while the European [20] and American guidelines [24] are less reluctant. However, patient comfort due to the reduced treatment time of hypofractionated RT and no increased late toxicity [1–4] should be considered.

Most of the radiation oncologists base their decision for or against additional pelvic LN irradiation in the definitive situation on suspect findings in imaging. Further, risk formulas and tables (e.g., Roach formula, Yale formula, Partin tables) are popular. The tumor stage, D’Amico risk group, and PSA dynamics play a subordinate role. Previous randomized controlled trials showed no benefit of additional pelvic RT for localized and locally advanced PC in the cN0 situation [6–9]. 26.6% of the participants state that in their institution, LN irradiation is not performed regularly. In the cN+ setting no randomized controlled data are available. However, several studies suggest that pelvic RT might have a positive impact on outcome [25–27]. Therefore, it seems valid to discuss pelvic RT in cases of positive LNs. Risk formulas and tables are still a valid aid to help assess the risk for microscopic nodal involvement. However, the emerging use of prostate-specific membrane antigen positron-emission tomography (PSMA-PET) imaging might replace such formulas in daily routine in the future.

Pelvic LN irradiation is performed with a median EQD2(1.5 Gy) of 47.52 Gy (range: 42.43–66.00 Gy). The dose prescriptions originate from previous trials on definitive RT and additional pelvic RT, with dose schemes of, e.g., 45 Gy in 1.8 Gy, i.e., EQD2(1.5 Gy): 42.43 Gy [28], or 50.4 Gy in 1.8 Gy, i.e., EQD2(1.5 Gy): 47.52 Gy [8]. However, the ideal dose for the LNs is still not clear.

Most participants will recommend additional ADT for patients with unfavorable intermediate-risk PC or higher. Only few radiation oncologists will exclusively start ADT for patients with high-risk PC or higher. Previous randomized controlled trials have shown that the addition of neoadjuvant or adjuvant ADT to definitive RT improves the outcome for patients with intermediate-risk and high-risk localized PC [29] as well as locally advanced PC [30]. Therefore, the European [31] and German [21] guidelines recommend additional ADT of 6 months for intermediate-risk localized PC and 36 months for high-risk localized/locally advanced PC. Recently, Nabid et al. showed that the outcome after 18 months of ADT for locally advanced PC is not different to 36 months of ADT with a higher quality of life in the short-term ADT group [32]. The trial was not designed as a noninferiority trial and results must be considered as speculative.

As for definitive RT, most participants use in-house SOPs for delineation of the PB. In terms of published guidelines, the RTOG [15] and EORTC [16] guidelines are those most used, while the PMH [17] and FROGG-RANZCR [18] recommendations are not widely implemented. Malone et al. compared all four guidelines and showed that the EORTC target is significantly smaller than the others, with limited inclusion of the anterior and superior sites [33].

The median EQD2(1.5 Gy) for postoperative RT of the PB is 66 Gy with range 60 to 74 Gy. The dose for salvage RT is not well defined. The European [31] and German [21] guidelines both recommend at least 66 Gy. For adjuvant RT no specific dose is mentioned in the guidelines [21, 31]. However, the prospective RAVES [34], RADICALS [35], and GETUG-17 [36] trials on early salvage versus adjuvant RT use the same 66 Gy for RT of the PB. Back in 2007, Goldner et al. showed that the dose for postoperative RT ranged from 60 to 72 Gy in Austria [22]. The SAKK 09/10 trial recently evaluated a dose escalation for salvage RT with a dose of 70 Gy in 2 Gy SD. Toxicity seems acceptable [37], although results of outcome must be awaited.

Most of the radiation oncologists base their decision regarding LN irradiation in postoperative RT (adjuvant or salvage RT) on suspect findings in imaging and postoperative tumor stage. In the adjuvant situation, patients receive RT based on the postoperative tumor classification. For adjuvant RT of the pelvic LNs, the European [31] and German [21] guidelines do not give clear recommendations in cases of positive LNs after radical prostatectomy (RP) and lymphadenectomy. Until now, no randomized clinical trials have been conducted on this matter. However, in a retrospective analysis, Abdollah et al. showed that patients with one to two positive pelvic LNs and adverse pathological findings (Gleason score 7–10, pT3b/pT4, or positive surgical margin) or patients with three to four positive pelvic LNs regardless of the pathological characteristics benefit from adjuvant RT [10].

For the salvage situation, the elective irradiation of pelvic LNs for patients with biochemical failure after RP remains a topic of discussion. However, the first results of the recently presented SPPORT trial (NRG Oncology/RTOG 0534) showed improved failure-free survival and reduced distant metastases for patients with RT of the PB and pelvic LNs plus ADT compared to RT of the PB with and without ADT [11].

The emerging use of PSMA-PET imaging gives the radiation oncologists the chance of targeting the morphologic correlate in cases of PSA rise after RP. In a previous series, we showed that PSMA-PET-based RT for patients with local recurrence and/or pelvic LN metastases (oligorecurrence in the pelvis) is feasible, with low toxicity and with an acceptable biochemical relapse-free survival of 74% [38].

Most radiation oncologists will recommend additional ADT to salvage RT for patients with risk factors. Of all participants recommending ADT, median duration is 12 months, with most of the radiation oncologists recommending 13–24 months (44.9%), followed by 6 months or less (34.8%). Two prospective and several retrospective trials investigated additional ADT to salvage RT [39]: Shipley et al. evaluated 24 months of bicalutamide for patients with salvage RT and stated an improved overall survival [40]. A post-hoc analysis revealed that additional ADT might even show better results for patients with risk factors such as Gleason score 8 to 10, PSA levels 0.7 to 4.0 ng/mL, or positive surgical margins. Carrie et al. evaluated 6 months of Goserelin and showed a benefit [41]. We did not acquire information on the ADT compound; however, the current state of the art of German-speaking radiation oncologists reflects the recently published data.

Re-RT after prior RT of the prostate or the PB is discussed controversially. Nearly 30% will not perform re-RT. In the literature, most re-irradiation was performed with focal low- or high-dose-rate brachytherapy, while only few series used SBRT for re-RT [42]. The 5‑year biochemical disease-free survival rates range from 20 to 77% for low-dose-rate brachytherapy and from 51 to 68% for high-dose-rate brachytherapy. For SBRT, the 2‑ and 3‑year disease-free survival rates ranged from 40 to 82% [42]. Cuccia et al. recently showed SBRT to be a safe and feasible treatment option for re-RT of local recurrence [43]. Overall, re-RT of the prostate or PB remains a treatment option for highly selected patients.

It has to be mentioned that our study has certain limitations, as do all online questionnaires. We did not distinguish between doses for salvage and adjuvant RT after RP to limit the length of the questionnaire. However, according to most guidelines, dose prescriptions are similar; therefore, we decided to omit this very specific question. For purposes of anonymity we did not document the country of origin. Therefore, we cannot make statements about regional differences. Further, no response rate can be presented due to the nature of online surveys via e‑mail. Our goal was to present the individual opinions of radiation oncologists, since it is inherent to online surveys that multiple answers from one institution cannot be prevented. The data reflect the day-to-day routine of German-speaking radiation oncologists; however, we consider this information as relevant and representative, which will be certainly applicable to other regions, too.

## Conclusion

Day-to-day patterns of care for patients with PC of German-speaking radiation oncologists are mainly in line with the published data. For target delineation, the use of published guidelines is widely spread. However, dose prescription is heterogenous for the prostate and PB, while doses of the pelvic LNs are mainly consistent. For definitive RT, most participants will irradiate pelvic lymph nodes in cases of suspect findings in imaging and based on risk formulas/tables. For postoperative RT, imaging and the postoperative tumor stage plays an important role. In case of definitive RT, most participants recommend additional ADT for unfavorable intermediate PC or higher. Most radiation oncologists recommend additional ADT for salvage RT. The presented data give an updated overview on treatment reality and might be used to sharpen future guidelines.

## Supplementary Information

Questions of the survey in German
