# Supplementary material for: Patterns of care for prostate cancer radiotherapy—results from a survey among German-speaking radiation oncologists
Source: Strahlenther Onkol. 2021 Jan 27;197(11):962–70. doi: 10.1007/s00066-020-01738-1 (PMC8547211; doi:10.1007/s00066-020-01738-1)
Supplement: Supplementary file 1 — Questions of the survey in German [file 66_2020_1738_MOESM1_ESM.pdf]

1. In welcher Einrichtung sind Sie tätig?
    a. Einzel- oder Gemeinschaftspraxis
    b. Medizinisches Versorgungszentrum (MVZ)
    c. Nicht-universitäre Klinik
    d. Universitätsklinik

2. In welcher Position sind Sie tätig?
    a. Arzt*Ärztin in Weiterbildung
    b. Angestellter*e Facharzt*ärztin
    c. Inhaber*in
    d. Medizinisch-technische*r Radiologieassistent*in
    e. Medizinphysik-Experte*in
    f. Anderes:

3. In welcher Position sind Sie tätig?
    a. Assistenzarzt*ärztin
    b. Facharzt*ärztin
    c. Oberarzt*ärztin
    d. Chefarzt*ärztin
    e. Medizinisch-technische*r Radiologieassistent*in
    f. Medizinphysik-Experte*in
    g. Anderes:

4. Welche Techniken stehen Ihnen zur Verfügung? (Mehrfachauswahl möglich)
    a. 3D-RT
    b. IMRT/VMAT
    c. Helikale IMRT
    d. IGRT
    e. Stereotaxie
    f. Protonen/Schwerionen
    g. Brachytherapie
    h. Andere:

Die folgenden Fragen beziehen sich auf die Planung und Durchführung einer definitiven Radiotherapie bei Patienten mit Prostatakarzinom.

5. Definitive Radiotherapie: Erfolgt zur Bestrahlungsplanung standardmäßig eine MRT-Untersuchung der Prostata?
    a. Ja
    b. Nein

6. Definitive Radiotherapie: Nach welchen Vorgaben wird das Zielvolumen der perkutanen definitiven Radiotherapie in Ihrer Einrichtung konturiert?
    a. Gemäß European Organization for Research and Treatment of Cancer (EORTC) Konturierungs-Empfehlung

   b. Gemäß European Society for Radiotherapy and Oncology Advisory Committee on Radiation Oncology Practice (ESTRO ACROP) Konturierungs-Empfehlung
   c. Interne SOP
   d. Individuell ohne Berücksichtigung der Konturierungs-Empfehlungen
   e. Andere:

7. Definitive Radiotherapie: Mit welcher Dosis wird die Prostata bei einer definitiven Radiotherapie behandelt?
   a. Gesamtdosis
   b. Einzeldosis

8. Definitive Radiotherapie: Wann werden die Lymphabflusswege in Ihrer Einrichtung mit behandelt (Mehrfachnennung möglich)?
   a. Aufgrund der PSA Dynamik
   b. Tumorstadium (cT)
   c. D'Amico Risikogruppen
   d. Nach Risikoeinschätzung (Roach Formel, Yale Formel, Partin Tabellen…)
   e. Bei auffälliger Bildgebung
   f. Die Lymphabflusswege werden nicht standardmäßig mit behandelt.
   g. Andere:

9. Definitive Radiotherapie: Mit welcher Dosis werden die pelvinen Lymphabflusswege bei einer definitiven Radiotherapie in Ihrer Einrichtung überwiegend behandelt?
   a. Gesamtdosis
   b. Einzeldosis

10. Definitive Radiotherapie: Wann empfehlen Sie Patienten bei der definitiven Radiotherapie eine zusätzliche Hormontherapie? (Mehrfachauswahl möglich)
    a. low-risk Patienten nach NCCN
    b. favourable intermediate-risk Patientennach NCCN
    c. unfavourable intermediate-risk Patientennach NCCN
    d. high-risk Patienten nach NCCN
    e. very high-risk Patienten nach NCCN

Die folgenden Fragen beziehen sich auf die Planung und Durchführung einer adjuvanten/Salvage-Radiotherapie bei Patienten mit Prostatakarzinom.

11. Postoperative Radiotherapie: Nach welchen Vorgaben wird das Zielvolumen der perkutanen Adjuvanten/Salvage-Radiotherapie in Ihrer Einrichtung konturiert?
    a. Gemäß Radiation Therapy Oncology Group (RTOG) Konturierungs-Empfehlung
    b. Gemäß European Organization for Research and Treatment of Cancer (EORTC) Konturierungs-Empfehlung
    c. Gemäß Australian and New Zealand Radiation Oncology Genito-Urinary Group (FROGG-RANZCR) Konturierungs-Empfehlung
    d. Gemäß Princess Margaret Hospital (PMH) Konturierungs-Empfehlung
    e. Interne SOP
    f. Individuell ohne Berücksichtigung der Konturierungs-Empfehlungen
    g. Andere:

12. Postoperative Radiotherapie: Mit welcher Dosis wird die Prostataloge bei einer perkutanen Adjuvanten/Salvage-Radiotherapie behandelt?

a. Gesamtdosis
b. Einzeldosis

13. Postoperative Radiotherapie: Wann werden die pelvinen Lymphabflusswege bei einer perkutanen adjuvanten/Salvage-Radiotherapie in Ihrer Einrichtung mitbehandelt? (Mehrfachauswahl möglich)
    a. Bei insuffizienter Lymphadenektomie
    b. Auf Basis der postoperativen Tumorklassifikation
    c. Bei auffälliger Bildgebung
    d. Initiale D'Amico Risikogruppen
    e. Nach initialer Risikoeinschätzung (Roach Formel, Yale Formel, Partin Tabellen…)
    f. Die Lymphabflusswege werden nicht standardmäßig mit behandelt.
    g. Andere:

14. Postoperative Radiotherapie: Mit welcher Dosis werden die pelvinen Lymphabflusswege bei einer perkutanen adjuvanten/Salvage-Radiotherapie überwiegend behandelt?
    a. Gesamtdosis
    b. Einzeldosis

15. Wann empfehlen Sie Patienten eine zusätzliche Hormontherapie im Rahmen der Salvage-Radiotherapie?
    a. In der Salvage-Situation erhalten die Patienten keine Hormontherapie
    b. Patienten mit Risikofaktoren (hoher PSA-Wert, Gleason Score, …) erhalten eine Hormontherapie
    c. Alle Patienten in der Salvage-Situation erhalten eine Hormontherapie

16. Für welchen Zeitraum (in Monaten) empfehlen Sie den Patienten eine zusätzliche Hormontherapie im Rahmen der Salvage-Radiotherapie?
    a. Monatsangabe
